# Inflammatory myofibroblastic tumor from molecular diagnostics to current treatment

**DOI:** 10.32604/or.2024.050350

**Published:** 2024-06-20

**Authors:** PAULINA CHMIEL, ALEKSANDRA SłOWIKOWSKA, ŁUKASZ BANASZEK, ANNA SZUMERA-CIEćKIEWICZ, BARTłOMIEJ SZOSTAKOWSKI, MATEUSZ J. SPAłEK, TOMASZ ŚWITAJ, PIOTR RUTKOWSKI, ANNA M. CZARNECKA

**Affiliations:** 1 Department of Soft Tissue/Bone Sarcoma and Melanoma, Maria Sklodowska-Curie National Research Institute of Oncology, Warsaw, 02-781, Poland; 2 Faculty of Medicine, Medical University of Warsaw, Warsaw, 02-091, Poland; 3 Department of Pathology, Maria Sklodowska Curie National Research Institute of Oncology, Warsaw, 02-781, Poland; 4 Department of Radiotherapy, Maria Sklodowska-Curie National Research Institute of Oncology, Warsaw, 02-781, Poland

**Keywords:** Inflammatory myofibroblastic tumor (IMT), Epithelioid inflammatory myofibroblastic sarcoma, Tyrosine kinase inhibitors (TKI), Anaplastic lymphoma kinase (ALK)

## Abstract

Inflammatory myofibroblastic tumor (IMT) is a rare neoplasm with intermediate malignancy characterized by a propensity for recurrence but a low metastatic rate. Diagnostic challenges arise from the diverse pathological presentation, variable symptomatology, and lack of different imaging features. However, IMT is identified by the fusion of the anaplastic lymphoma kinase (ALK) gene, which is present in approximately 70% of cases, with various fusion partners, including ran-binding protein 2 (RANBP2), which allows confirmation of the diagnosis. While surgery is the preferred approach for localized tumors, the optimal long-term treatment for advanced or metastatic disease is difficult to define. Targeted therapies are crucial for achieving sustained response to treatment within the context of genetic alteration in IMT. Crizotinib, an ALK tyrosine kinase inhibitor (TKI), was officially approved by the US Food and Drug Administration (FDA) in 2020 to treat IMT with ALK rearrangement. However, most patients face resistance and disease progression, requiring consideration of sequential treatments. Combining radiotherapy with targeted therapy appears to be beneficial in this indication. Early promising results have also been achieved with immunotherapy, indicating potential for combined therapy approaches. However, defined recommendations are still lacking. This review analyzes the available research on IMT, including genetic disorders and their impact on the course of the disease, data on the latest targeted therapy regimens and the possibility of developing immunotherapy in this indication, as well as summarizing general knowledge about prognostic and predictive factors, also in terms of resistance to systemic therapy.

## Introduction

### Epidemiology

An inflammatory myofibroblastic tumor (IMT) is a rare mesenchymal tumor that was first reported in 1939 and easily misdiagnosed as a highly malignant sarcoma [1]. It comprises differentiated myofibroblastic spindle cells with numerous plasma cells and/or lymphocyte infiltrates. According to the World Health Organisation (WHO) classification, IMT is a low-grade or borderline mesenchymal tumor [2]. IMT staging with the Pathologic Soft Tissue Stage Classification (pTNM; AJCC 8th Edition) is recommended, where IMT is classified according to the anatomical location of the primary tumor-head and neck, trunk and extremities, abdomen and thoracic visceral organs, retroperitoneum, and orbit [3,4]. This approach helps IMTs management optimized for aforementioned anatomical manifestations [2]. The most common IMT manifestation is the lung [5–7]. It also presents a strong predilection for visceral organs and deep soft tissues of the abdomen, pelvis, retroperitoneum [8–11], head, and neck [12,13], but any part of the human body may be affected, including somatic soft tissues, bone, extremities, larynx, or even central nervous system [9,14]. Some rare locations include the esophagus [15], the pericardium [12], the heart [7], the spinal meninges [15], and the adrenal glands [16,17]. A case describing the simultaneous location of IMT lateral to the ascending aorta near the root and inside the atrial septum has been reported [18]. IMT may also occur in pregnant women, potentially leading to pregnancy complications and worsening symptoms after delivery [19]. In addition, there have been very rare cases of IMT within the spermatic cord in elderly men, as described in the literature [6]. In rare cases, IMT may be located in the subglottis area or within the vocal cords, causing breathing disorders [20,21]. One of the youngest patients described in the literature, a 5-month-old boy [22], presented with IMT located in the orbit. Spleen tumors, among other infantile forms, have been reported to cause growth restriction in infants [23]. A case of coexistence of Wilms’ tumor with IMT has also been reported, which raises the question of whether these tumors share a common pathogenesis or whether IMT could have been induced by treatment of Wilms’ tumor [24]. IMT development occurs at all ages with most patients diagnosed below 40 years of age [25–27], although it can develop as late as the eighth decade of life [28,29]. It is diagnosed with a slight predominance of females to men [30,31].

### Clinical features

The clinical manifestations of IMT are closely related to the site of the primary tumor. Known IMT symptoms are pain [32], fever [33,34], decreased appetite [34,35], weight loss [33,36], fatigue, and malaise [28]. Pulmonary IMT can cause productive or dry cough [34], chest pain [29], dyspnea [14], and hemoptysis [37,38]. At diagnosis, pulmonary IMT is associated with upper respiratory infection or pneumonia in 30% of patients [39,40]. Tumors located in the abdomen and pelvis may cause symptoms that vary according to the location: nausea, vomiting [41,42], dysphagia [43], reflux [36,44], melaena [45], abdominal or back pain [16,42], hematuria [46], menorrhagia [47] or even gastrointestinal obstruction [48]. Tumors range from 1 to >20 cm in maximum dimension [10,26,30,37,41,49–51], with a mean size of 4–8 cm [11,27,52,53].

### Prognosis

In general, IMT has a benign course with favorable results after radical treatment. Distant metastases are rare, occurring in <5% of cases [27,31,33,54]. A recent study of 92 patients with IMT confirmed a metastatic rate of approximately 5%, as 5 of 92 patients experienced metastases at presentation [55], which is consistent with other reports in the literature [11,33]. IMT with atypical pathology or exhibiting an aggressive clinical course revealed greater metastatic potential, with 10.2% of patients showing distant metastases [53]. In particular, metastatic cases were reported to be limited to tumors with downregulated anaplastic lymphoma kinase (ALK), as none of the ALK-positive patients exhibited metastases. The most common sites of metastasis are the lung and brain, followed by the liver and bone [27,33]. Less common localizations include abdomen and mesentery, retroperitoneum, mediastinum, neck, and forearm [29,42,52–54,56–58]. Metastases are usually identified at the time of initial diagnosis or within one year of diagnosis [12,31,33,57,59], but patients develop metastases more than 10 years after the excision of the primary tumor [27,33,34,60]. IMT shows a recurrence rate of approximately 25%; however, this rate varies according to the anatomical site, and lung tumors are the least prone to recurrence, occurring in approximately 2% of cases [9,61,62]. In the pediatric population, 21% of the patients experienced recurrence, including 2 patients who died from the disease [63]. Observations of aggressive intraabdominal cases indicate that the recurrence rate may be higher, as all patients who underwent radical operations exhibited extremely rapid disease recurrences [64]. Recurrences after radical treatment are associated with a worse prognosis [65].

## Diagnosis

The wide spectrum of non-specific symptoms leads to difficulties in IMT diagnostics. Untypical and nonspecific symptoms and imaging findings make it difficult to differentiate it from other neoplasms [31,36]. The diagnosis is difficult preoperatively; Most tumors are diagnosed after resection. An appropriate histological diagnosis of IMT preoperatively or intraoperatively is one of the main problems; needle biopsy has been suggested as an optimal diagnostic approach, but multiple areas of the tumor need to be evaluated [32]. IMT is frequently discovered incidentally during routine examinations or when investigating other medical conditions. In routine clinical practice, computed tomography (CT) and magnetic resonance imaging (MRI) constitute the fundamental diagnostic modalities [66]. However, specific imaging characteristics for IMT are lacking, and radiological findings typically reveal solid, regular, well-defined masses, contingent upon the primary tumor’s location [67]. In general, in contrast-enhanced computed tomography (CT) IMT shows homogeneous or heterogeneous lesions, exhibiting various morphologies ranging from infiltrative masses to well-delineated masses with inflammatory and fibrotic components [68–70]. IMT tumors present with mild to moderate enhancement and delayed uptake can occur due to the presence of fibrosis [70]. Calcification is generally absent, although its presence may suggest alternative malignancies [70,71]. In magnetic resonance imaging (MRI) IMT has low signal intensity in T1 and T2 weighted images, hyperintensity on T2 scans, and more obvious enhancement indicating rapid growth and increased aggressiveness [70,72,73]. In addition, peritumoral edema can be found in MRI, indicating a benign course and helping to define surgical margins [70]. In particular, specific tumor locations can exhibit unique characteristics in imaging studies. Neoplasms located in bones may cause destruction [69]. IMT in the lungs is most often located peripherally, showing a slight contrast enhancement, and in some cases, the presence of calcification has been demonstrated [71,74]. IMTs located in the urinary bladder are typically found as submucosal masses, characterized by a generally elevated T2 signal on MRI and heterogeneous contrast enhancement [75]. 18F-fluorodeoxyglucose positron emission tomography/computed tomography (18F-FDG PET/CT) provides additional information on the diagnosis and differentiation of IMT. A study involving 5 patients revealed a heterogeneous standardized uptake value (SUVmax) of approximately 10.9, with this value being predominantly influenced by tumor cellularity, nuclear atypia, and a relatively high proliferative index [59]. However, SUVmax values for IMT vary significantly, ranging from 1.84 to 28.6, depending on the specific case [76–78]. The utility of this diagnostic tool extends to the detection of metastatic disease and the monitoring of response to treatment [79]. A case report highlighted two distinct IMT states; the first conducted before the initiation of therapy showed an SUVmax of 15.1. Subsequently, after 3 weeks of steroid treatment, SUVmax decreased to 5.4, indicating a favorable metabolic response to therapy [80]. 68Ga-DOTANOC PET-CT has shown utility in tumors expressing somatostatin receptors, but there are new reports indicating the utility of this diagnostic tool in IMT. The case report showed the usefulness of this test in the differential diagnosis of IMT, especially those located in the gastrointestinal tract [81]. The tests mentioned above can guide the differential diagnosis towards IMT; however, initial misdiagnosis is often the case. The differential diagnosis should predominantly consider other primary malignant neoplasms, as well as benign diseases such as hematoma, lipoma, or granulomatosis (sarcoidosis, Wegener’s), and infectious diseases such as tuberculosis and pyogenic infections [8,11,13,36,82]. Distinguishing IMT from non-neoplastic systemic inflammatory diseases is crucial. Historically, the term inflammatory pseudotumor (IPT) encompassed masses arising from various connective tissue cells and inflammatory infiltrates. However, the contemporary classification separates IMT from this broad group of diseases. In differential diagnosis, attention should be focused on specific characteristics such as older patients’ age, absence of genetic disorders, and increased lymphoplasmocytic infiltration within the tumor in the case of IPT [83]. A different subtype of IPT is IgG4-related IPT, which differentiates further from IMT based on a markedly elevated percentage of IgG4 antibodies in the patient’s blood [84]. Emerging diagnostic markers have been evaluated, however, the data is contradictory and lacks confirmation in large prospective trials. In immunohistochemistry, IgG4 plasma cells have shown effectiveness in differentiating IMT from IPT. IPT has shown denser infiltration of these cells within the tumor compared to IMT (mean, 127.8/high-power fields *vs*. 17.8/high-power fields) [85]. It is important to note that these findings are not conclusive and require further investigation. Other studies have also confirmed this observation, indicating that the IgG4/IgG ratio ≥ 0.10 found in both IPT and IMT cases is insufficient for differentiation in independent analysis [86]. Additionally, cancer stem cells were found within the lung IMT, which may serve as an additional marker in pathological and differential diagnosis in the future [87]. To distinguish it from other malignant lesions, particular attention should be paid to differentiation from inflammatory fibrosarcoma [88], gastrointestinal stromal tumor (GIST) [89], and desmoid fibromatosis [90]. Currently, differentiation among these entities is based on a thorough pathological examination and the identification or absence of specific genetic markers within the tumor with next-generation sequencing (NGS) [91].

## Pathology

### Microscopic presentation

IMTs present a broad morphologic spectrum, from inflammatory lesions to sarcomatous neoplasms [12,92]. Microscopically, IMTs exhibit a varied cellularity with bland-appearing proliferation of spindle cells arranged in fascicles in a myxoid to collagenous stroma accompanied by diverse inflammatory components [9–11,33,93]. These spindle cells predominantly resemble myofibroblasts and contain a minor fibroblast component, with about half of the cases displaying scattered ganglion-like cells [11,33]. Three basic histological patterns can be identified within the same tumor, typically with one of the patterns being predominant [11,33,94]. The first pattern, myxoid/vascular, features loosely arranged rounded spindle cells in an edematous/myxoid stroma with a pronounced vascular component, and comparatively to other patterns fewer plasma cells, neutrophils, and eosinophils [11]. The second pattern, compact spindle cell pattern cells are plump to gangliocytic myofibroblasts, arranged in fascicles or storiform, in a collagenous, myxoid, loose stroma, densely populated with inflammatory cells such as plasma cells and lymphocytes. The inflammatory infiltrate is mixed with spindle cells, lymphoid follicles, and aggregates of plasma cells that can also be seen [11,52]. The third pattern, hypocellular fibrous, also known as fibromatosis-like pattern, is hypocellular with elongated spindle cells in the dense, collagenous stroma and scattered inflammatory cells-lymphocytes, plasma cells, and eosinophils, mitoses can be found, and dystrophic calcifications can also be seen [11,33,52].

IMTs are generally not associated with calcification, vascular invasion, hemorrhage, or necrosis, although focal dystrophic calcification and metaplastic ossification may occur [11,52]. Morphologically, IMT can become a more severe lesion of variable appearance. Cellular atypia, which can indicate recurrence or malignant transformation, does not significantly differ in cases with or without these conditions, based on cellularity, mitotic activity, and inflammatory infiltration [11,54]. Significant inflammatory infiltration predominantly comprises abundant plasma cells and lymphocytes, occasionally eosinophils and neutrophils [10,11,33,93].

### Immunohistochemistry

**Figure 1:**
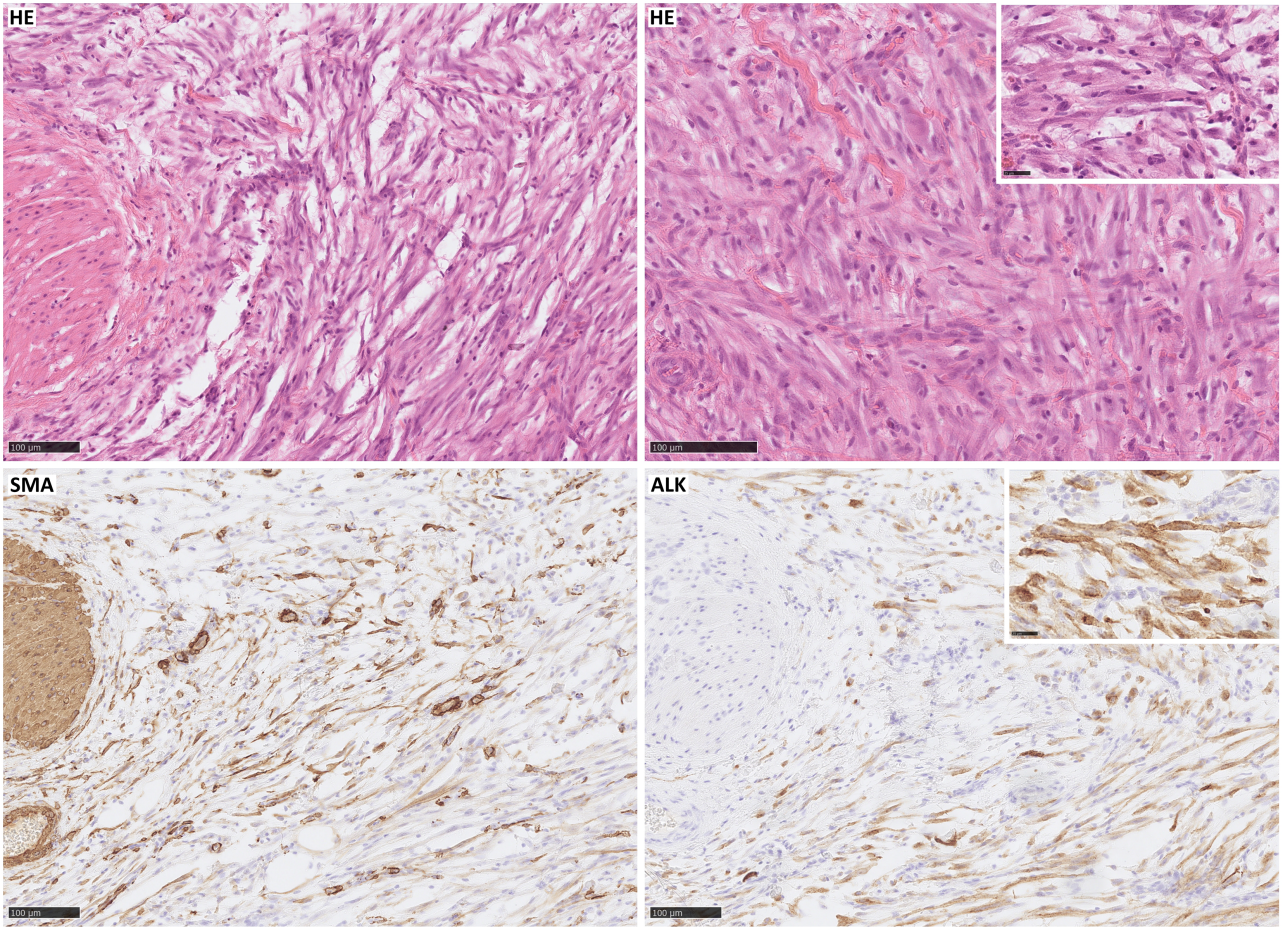
Inflammatory myofibroblastic tumor with typical morphology: spindle cells mixed with a scant inflammatory background (HE); immunohistochemical expression of SMA and strong expression of ALK (the case was genetically confirmed). Scale bar, 100 µm.

Immunohistochemically, IMT presents the features of myofibroblastic differentiation [9], with variable reactions to muscle markers [52]. The expression of smooth muscle actin (SMA) is noted in 80%–90% of spindle cells [10,12,34,36,44,49,95–99], while muscle-specific actin (MSA) [100], desmin, and calponin are seen in 60%–70% of cases, often focally [11,12,30,46,49,60,94–102]. Vimentin staining in spindle cell cytoplasm is typically strong and diffuse [41,44,46,49,93,95,99]. About 30% of tumors show focal cytokeratin reactivity [11,49,52,96–102]. ALK reactivity is seen in 36%–73% of cases, mostly strong, but dependent on the fusion partner (diffuse cytoplasmic, perinuclear, granular cytoplasmic, nuclear membranous immunoreactivity) [12,29,33,34,38,41,46,52,93,95,96,100–103], correlating with local recurrence rather than distant metastases. Also, ALK-positive tumors are found mostly in younger patients, with a predilection for patients under 40 years of age [29,53,94]. Nuclear p53 expression is noted in 80% of IMTs, but only 25% in metastatic cases [53]. Various markers such as CD34, EMA, CD68, CD10, keratin, laminin, and fibronectin show positivity [49,95–97,99,100], with partial positivity for p16 and WT-1 [36]. Cytokeratin and epithelial membrane antigens were detected mainly in the airways [99]. Cells are most often negative for markers like B-catenin, S100, myogenin, myoglobin, CD68, CD34, c-kit, CD21, CD35, CD117, and caldesmon (Fig. 1) [49,94,99,101,104,105].

### Tumor microenvironment

Typically, IMT contains a significant inflammatory infiltrate, mostly composed of plasma cells (CD138 positive) and abundant lymphocytes-T (CD3 positive) and B (CD20 positive) [10,47,96,100,106]. Genetic analysis of many types of STS has shown that IMT has one of the highest T cell inflamed scores (TIS), which indicates the immunologically active tumor microenvironment influencing the possible response to immunotherapy [107]. Eosinophils, neutrophils, macrophages (CD68 positive), and histiocytes occur sporadically [10,11,36,96,99,108]. However, the spectrum of morphology may be vast, depending on the histological pattern of IMT [12,33].

### Differential diagnosis

Par excellence, IMT can mimic nodular fasciitis, fibrous histiocytoma, desmoid fibromatosis, desmoid tumor, scar, calcifying fibrous tumor, myofibromatosis, fibrosarcoma, leiomyoma, leiomyosarcoma, rhabdomyosarcoma, and mediastinal fibrosis, occasionally spindle cell sarcoma, spindle cell melanoma, sarcomatoid carcinoma and inflammatory fibrosarcoma and fibromyxoid sarcoma, making differential diagnosis extensive [11,28,32,33,40,44,46,47,57,58,96,102,109–112]. In the gastrointestinal tract and mesentery, the differential diagnosis includes GISTs, inflammatory pseudotumors, proliferative fasciitis, dedifferentiated liposarcoma, and other mesenchymal tumors due to histological similarities [29,33,52,105]. In the lungs, malignant lymphoma, lymphoid hyperplasia, pseudolymphoma, plasmacytoma, sarcomatoid carcinoma, sclerosing hemangioma, nodular pneumonitis, fibrosis, organized pneumonia, mesenchymal neoplasms (various histotypes of sarcoma), must be considered [26,27,33,38,49]. ALK-negative IMTs pose a diagnostic challenge, particularly with conditions like sclerosing mediastinitis, IgG4-related disease, MALT lymphoma, hyalinizing granuloma, and spindle cell sarcomas with a myofibroblastic or fibroblastic component [86,92]. The absence of certain features such as anaplasia, spindle cells admixed with plasma cells and lymphocytes, the lack of atypical mitosis and mitotic figures, the paucity of nuclear hypochromasia, and necrosis or vascular invasion help distinguish IMTs from many types of carcinomas [32,33]. Immunochemistry helps identify myofibroblastic markers and ALK expression, but the absence does not exclude IMT (Table 1) [33].

**Table 1:** Immunohistochemical characteristics are useful in the differential diagnosis of inflammatory myofibroblastic tumor (IMT) differential diagnosis

Diagnosis	Immunohistochemical features							References
	ALK	CD117	Desmin	SMA	CK AE1/AE3	S100	EMA	
Inflammatory myofibroblastic tumor	+/−	–	+/−	+	+/−	–	−/+	[11,53,61]
GIST	–	+	–	+/−	−/+	–	−/+	[29,105]
Rhabdomyosarcoma*	−/+	–	+	+	+/−	–	–	[102,113]
Leiomyosarcoma	–	–	+	+	–	–	+/−	[94]
Schwannoma**	–	–	–	–	−/+	+	−/+	[33]
Solitary Fibrous Tumor***	–	–	–	−/+	–	–	−/+	[114]
Fibromatosis****	–	–	–	+/−	–	–	–	[62]
Squamous cell carcinoma^#^	–	–	–	–	+	–	+	[98,115–117]

Note: ALK-anaplastic lymphoma kinase; CD117-differentiation 117; SMA-smooth muscle actin; CK AE1/AE3-Cytokeratin AE1/AE3; EMA-epithelial membrane antigen; *MyoD1 and myogenin positivity supports diagnosis; **Strong expression of S100 and SOX10 is required; ***STAT6 nuclear immunohistochemical expression or identification of STAT6 rearrangement in fluorescence *in situ* technique is important for the final diagnosis; ****B-catenin nuclear expression favors the fibromatosis diagnosis; ^#^in routine diagnostics, expression of p40, p63, and CK 5/6 are typical for squamous cell carcinoma.

## Genetics and Molecular Biology

### ALK alterations in cancer

**Figure 2:**
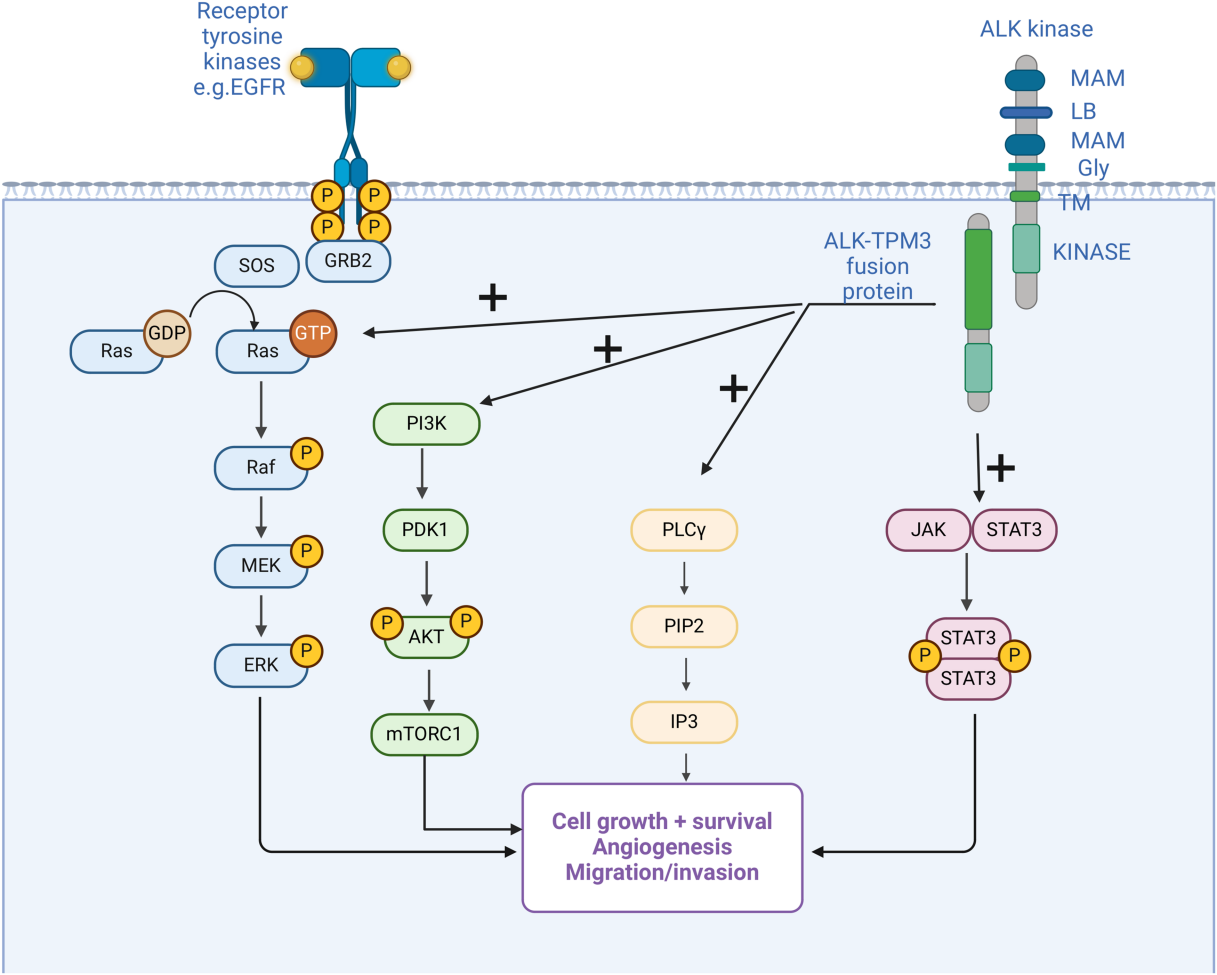
Anaplastic lymphoma kinase (ALK) fusion in inflammatory myofibroblastic tumor (IMT) and the main activated oncogenic pathways. Structure of ALK with two meprin A 5 proteins, receptor protein tyrosine phosphatase μ regions (MAM), glycine-rich domains (GR), a low-density lipoprotein motif (LDL), and an intracellular domain of tyrosine kinase [126]. Fusion with multiple possible partners leads to ligand-independent activation of downstream pathways, which are also activated under physiological conditions by other mechanisms. Activation of oncogenic pathways results in cancer initiation and progression by the mechanisms mentioned in the figure.

In 1994, anaplastic lymphoma kinase (ALK) was initially identified as a tyrosine kinase in cell lines of anaplastic large cell lymphoma (ALCL), thus deriving its name from this association [118,119]. Since its initial discovery, extensive investigations have been conducted to elucidate the structural, origin, and functional aspects of the ALK receptor. The human ALK gene is located in the chromosome region 2p23.2–p23.1 and consists of 26 exons that encode the full-length ALK protein that includes 1620 amino acids [120,121]. ALK, a tyrosine kinase enzyme, belongs to the receptor tyrosine kinase (RTK) family [122,123]. The inherent function of this enzyme primarily involves catalyzing the phosphorylation of tyrosine residues on substrate proteins and activating various cellular mechanisms and oncogenic pathways. Currently, two physiological ligands for ALK have been identified as FAM150A (ALKAL1 or Augmentor α) and FAM150B (ALKAL2 or Augmentor β) [124]. The role of the protein in the nervous system and intestinal development has been extensively documented, with predominant expression of ALK mRNA observed in the brain, small intestine, and colon [125,126]. Activation of ALK alterations, which include mutations, amplifications, and fusions/rearrangements, has been identified in various malignancies [127]. A comprehensive cross-cancer analysis estimates that approximately 3.3% of cancers harbour ALK alterations, with ALK fusions detected in approximately 0.5%–0.8% of all cancers [128]. Cancers harbouring ALK fusion are mainly lung tumors, but also brain tumors, thyroid cancers, sarcomas, and gastrointestinal cancers. The most frequently occurring fusion partners are EML4 and STRN, while PPP1CB-ALK, NUP107-ALK, COL14A1-ALK, BRAF-ALK and RASD2-ALK fusions are rarely observed [129]. Fusions and rearrangements induce ligand-independent activation of ALK, leading to sustained activation and stimulation of four major oncogenic pathways: janus kinase-transcription signal transducers and activators (JAK-STAT), mitogen-activated protein kinase/extracellular signaling-regulated kinase (MAPK/ERK), phospholipase C gamma (PLCγ), and phosphatidylinositol-3-kinase–protein kinase B (PI3K-Akt) (Fig. 2) [130–132]. These pathways are essential in cell cycle progression, proliferation, apoptosis, angiogenesis, and cell survival [133].

### ALK alterations in IMT

Approximately 50%–70% of IMT show ALK rearrangements [12,33,34,37,41,134], in the short arm of chromosome 2 in the p21-p23 region p21-p23 [101,103], with prevalence depending on the primary tumor location-most of the lung, then GI and liver, bladder, intrabdominal, trunk, and mediastinum, head and neck [12]. In IMT, immunohistochemistry (IHC)-assessed ALK expression reliably predicts the presence of an ALK rearrangement (detected by fluorescence *in situ* hybridization-FISH or reverse transcription polymerase chain reaction-RT-PCR) [103,135] and the pattern of ALK immunostaining and localization within the cell appears to be determined by specific gene fusions [33]. Several ALK fusion partners have been identified, including TMP3 at 1p23, TPM4 at 19p13, ATIC at 2q35, CLTC at 17q23, CARS at 11p15, RANBP2 at 2q13 and SEC31L1 at 4q21 [46,62,136–139] and also FN1, EML4, PRKAR1A, TNS1, LMNA [46,51,98,140]. EML4-ALK is one of the most common fusion variants in thoracic IMT compared to other anatomic sites [12,92]. Furthermore, the aggressive subtype of IMT, i.e., epithelioid IMT (eIMT), harbors characteristic fusion with RANBP2 in numerous cases [141,142]. In addition, fusions with RANBP1 and EML4 have been described in eIMT [143,144]. Genetic analysis of tissues from participants in the CREATE study yielded additional insights into potential fusions in IMT. Among the 24 samples examined, 20 exhibited consistent results in IHC and FISH tests. Sixteen ALK (+) tumors with distinct fusion partners were identified and one mutation affected other genes. The identified fusion partners included genes such as TPM3, CARS, LRRFIP, TNS1, RANBP2, IGFBP5, NRP2, SQSTM1, ATIC, KIF5B, LRRFIP, and EML4 [145]. However, advances in recent years, coupled with the accessibility of genetic testing, have revealed that the previously identified options represent only a subset of the potential variations observed in IMT. Numerous case reports suggest the existence of a broader spectrum of disorders involving the ALK gene, as defined in Table 2.

**Table 2:** Case studies on the use of various ALK inhibitors in individuals with advanced/metastatic IMT

Reference	Targeted therapy*	ALK status	Other treatment	Response to treatment	Patient information (age, sex, primary tumor site)
[189]	Crizotinib 500 mg/day	RANBP2-ALK rearrangement	Surgery	PR	22; male; pelvis with peritoneal dissemination
[190]	Crizotinib	ALK rearrangement	–	CR	45; female; liver; metastatic
[191]	Crizotinib 250 mg/2 times a day	RANBP2-ALK rearrangement	Surgery, chemotherapy with doxorubicin	CR	22; male; pelvis
[192]	Crizotinib	CLTC-ALK rearrangement	–	SD	24; male; omentum, liver, colon
[193]	Crizotinib 250 mg on alternate days	DCTN1-ALK rearrangement	Pazopanib	PR	50; female; uterine
[194]	Crizotinib 200 mg/twice daily	–	–	CR	7; female; right eye
[195]	Crizotinib 250 mg/2 times a day	EML4-ALK rearrangement	Non-steroidal anti-inflammatory drugs, steroids, vinblastine, combination chemotherapy with alternate VAC/VA, and vinorelbine and methotrexate, palliative radiotherapy	CR	16; female; right arm
[196]	Crizotinib	RANBP2-ALK rearrangement	6 cycles of chemotherapy in 4 was gemcitabine with docetaxel	PR	15; female; ovary; recurrence
[82]	Crizotinib 250 mg/2 times a day	NUMA1-ALK rearrangement	Nivolumab at 3 mg/kg intravenously every 2 weeks	CR	21; female; left arm; metastatic
[106]	Crizotinib 250 mg/2 times a day	LRRFIP1-ALK rearrangement	–	PR	15; male; hip
[197]	Crizotinib 500 mg daily	ALK rearrangement	Surgery	CR	37; female; right adrenal gland and inferior vena cava; metastatic
[198]	Crizotinib	ALK wt	–	PR	31; male; lung; metastatic
[199]	Crizotinib 250 mg/2 times a day	IGFBP5-ALK-Rearrangement	Surgery	CR	56; female; uterus; recurrence
[200]	Crizotinib 280 mg/m^2^/day	CLTC-ALK rearrangement	–	CR	8-months; male; common bile duct and celiac artery
[201]	Alectinib 600 mg/day	SQSTM1-ALK rearrangement	Surgery	PR	31; female; Recurrence of multifocal, unresectable multifocal disease
[202]	Ceritinib 300 mg/m^2^/day	ALK rearrangement	Surgery, nonsteroidal anti-inflammatory drugs, chemotherapy with methotrexate and vinorelbine	CR	17 years old; male; relapse with lung metastasis
[203]	Alectinib	TNS1-ALK rearrangement	Surgery, 6 cycles of olaratumab (a PDGFR-alpha blocker) and doxorubicin	CR	71; female; uterus; recurrence
[31]	Alectinib 600 mg/day	SQSTM1-ALK rearrangement	–	PR	46; male; unresectable recurrence
[204]	Ensartinib 225 mg/day	RANBP2-ALK rearrangement	–	PR	31male; metastatic recurrence
[205]	Alectinib 600 mg/day	EML4-ALK rearrangement	–	PR	26; male; left forearm; metastatic
[206]	Alectinib 600 mg/day	EML4-ALK rearrangement	Surgery	PR	55; male; metastatic
[207]	Crizotinib Ceritinib to 600 mg daily	TPM3-ALK rearrangement	Surgery, celecoxib	PR	32; male; left lung and chest wall, medial right thigh, right gluteal muscle, and omentum
[208]	Crizotinib Ceritinib 750 mg/daily	ALK G1269A mutation	Surgery	PR	36; female; lung; recurrence
[209]	Crizotinib Ceritinib 750 mg/daily Alectinib 600 mg/daily Lorlatinib 100 mg/daily	ALK rearrangement	–	PR	18; female; brain; metastatic
[210]	Crizotinib Brigatinib 180 mg/daily Lorlatinib 180 mg/daily	Rearrangement of the TFG-ROS1 rearrangement	Surgery	CR	14; female; brain; metastatic
[211]	Crizotinib 250 mg/twice daily Alectinib 600 mg/twice daily Ceritinib 450 mg/daily Lorlatinib 100 mg/daily	PRRC2B-ALK rearrangement	Surgery	PR	42; female; pelvis; recurrence
[212]	Crizotinib 250 mg/twice daily Ceritinib 450 mg/daily Alectinib 600 mg/twice daily	RRBP1-ALK rearrangement	–	PR	22; male; abdomen; metastatic

Note: *In the studies referenced, the doses administered exhibited variability throughout the therapeutic course, mainly due to drug-related toxicities. We presented the initial or principal dose used among the patient cohort. CR-complete response, PR-partial response, SD-stable disease, ALK-anaplastic lymphoma kinase, RANBP2-ran binding protein 2, CLTC-clathrin heavy chain, RRBP1-ribosome-binding protein 1, PRRC2B-proline-rich coiled-coil 2B, DCTN1-dynactin subunit 1, EML4-echinoderm microtubule-associated protein-like 4, NUMA1-nuclear mitotic apparatus protein 1, LRRFIP1-leucine-rich repeat of flightless-1 interacting protein 1, IGFBP5-insulin-like growth factor-binding protein 5, SQSTM1-sequestosome 1, TNS1-tensin 1, TPM3-tropomyosin 3 TFG-tropomyosin-receptor kinase fused gene, ROS1-proto-oncogene 1 receptor tyrosine kinase, PDGFR-platelet-derived growth factor receptor, VAC/VA-vincristine, actinomycin-D, and cyclophosphamide.

Patients with ALK abnormalities are younger, with a slight male predominance, with a similar anatomic tumor distribution and greater recurrence [103]. As we know, there exists a cohort of patients who are ALK (−) and frequently exhibit disorders in other genes that activate comparable signaling pathways within cells.

### Other genetic alterations in IMT

Another common gene fusion includes ROS1 and RET gene fusions [12,146]. Fusion of the ROS1 gene is the second most common abnormality detected in thoracic IMT after rearrangement of the ALK gene [92]. In IMT, there is a subgroup of patients harbouring NTRK fusions [147], as studies showed this is a prevalent mutation, present in 18% of ALK (−) patients [148]. The most common partners are NTRK1 and NTRK3 and the most frequent fusion is ETV6-NTRK3 [149]. The patient mentioned above from the CREATE trial had an exact ETV6–NTRK fusion [145]. Furthermore, the NTRK fusion case showed a response to crizotinib [150]. Other less common fusions involve PDGFRβ, RET, NTRK, and IGF1R [51,151–153].

### Diagnostic implications

IHC has the potential to provide information on these genetic rearrangements. IHC has proven useful, for example, in the diagnosis of NTRK fusions, as they show constant nuclear and cytoplasmic staining for pan-Trk in the majority of tumor cells [154]. It is important to emphasize that, while IHC staining can reveal details about the phenotype, it does not directly yield data regarding genotype. Consequently, discrepancies can arise between the observed phenotype and the actual genotype. Some IHC-positive IMTs show no evidence of ALK rearrangement in FISH, which is false positive [53,155]. Negative FISH result should not necessarily exclude a diagnosis of IMT in the setting of typical morphology, particularly when tumor cells are positive for ALK or ROS1 by IHC, in either situation additional molecular tests such as Archer or other targeted RNA NGS sequencing panel is required to confirm the presence of genes fusion [92]. Next generation sequencing and determining the exact mutation outside the diagnosis may also be useful in choosing treatment, so it is worth adding this element to the evaluation of patients with IMT [156,157]. However, both IHC and FISH can be reliably used to detect gene rearrangements including ALK, ROS1, PDFGRB, NTRK1, RET, EML4, TFG, and TMP4 [12,33,92,93,101,113,54].

EGFR-Epidermal growth factor receptor, ERK- extracellular signal-regulated kinase, GRB-growth factor receptor-bound, GTP/GDP-guanosine triphosphate/diphosphate, IP3-inositol triphosphates, JAK-Janus kinase, MEK-mitogen-activated protein kinase, rapamycin-mTOR-mammalian target, PDK1-pyruvate dehydrogenase kinase 1, PI3K-phosphoinositide 3 kinase, PIP2-phosphatidylinositol 4,5-bisphosphate, PLC-γ-phospholipase C γ, Raf-rapidly accelerated fibrosarcoma, Ras-rat sarcoma virus gene, SOS- son of sevenless gene, STAT signal transducer and transcription activator, TPM 3-tropomyosin 3. Created by Biorender.com.

## Surgical Treatment

According to the 2021 European Society for Medical Oncology (ESMO) guidelines (Gronchi et al.), tumor surgery must be performed by a surgeon trained in sarcoma surgery and preferably within a sarcoma center [158]. Due to the rarity of the disease and the lack of large cohort analysis, most of the knowledge on surgical treatment of IMT comes from case reports or case series. Depending on tumor size and anatomy, total surgical excision (TSE) or wide local excision (WLE) remains a first-line treatment for IMTs for as long as a complete resection involving removal of the tumor with an envelope of surrounding healthy tissue can warrant complete radical excision (R0). According to Sagar et al. and Iwai et al., complete excision of the tumor has a good prognosis, with a 5-year survival rate of 91% [159,160]. Following complete excision, the rate of recurrence for IMT can vary depending on the anatomical location. It can range from 2% for tumors limited to the lung and up to 25% for tumors in extrapulmonary locations. Multinodular intraabdominal tumors are prone to the highest recurrence rate, as well as IMTs that are surgically challenging anatomical locations such as the head and neck region where complete surgical resection can pose great difficulty for a surgeon. Statistically, en-bloc excision with clear margins (R0) of a single IMT has a very low recurrence rate [27,61,33,161–164]. Several studies advocate for re-excision of local recurrence as a treatment of choice, with little evidence available for optimal therapy for inoperable, relapsed, or metastatic IMT [11,162]. Some articles advise a flexible approach to IMT resection margins and suggest that a final decision on an aggressive approach to tumor resection should be made after the intraoperative frozen section is performed and a report of the injury is provided [165]. Anteby et al. reported a case of local recurrence and distant site metastases after the removal of primary IMT from the gallbladder. The central tumor mass was found in the gallbladder, with local spread to the liver and the surface of the duodenal wall and pancreas. An intraoperative pathology report of the frozen section of the gallbladder did not confirm the malignancy of the tumor, but only inflammatory changes were found. A non-radical resection was performed to avoid a complex and high-risk Whipple procedure. Unfortunately, this resulted in a large local recurrence within a year of the initial surgery. The patient underwent a second procedure with negative margins but was followed by a lung disease shortly after. This particular case draws attention to the possibility of micrometastasis and raises the question of possible adjuvant systemic therapy for tumors that exhibit aggressive behaviour [166]. Long-term follow-up after radical and nonradical excision is mandatory in all cases diagnosed as IMTs.

## Radiotherapy

### Neoadjuvant and adjuvant radiotherapy

The role of perioperative radiotherapy in IMT has not been established. However, it should not be considered as the standard of care due to lack of evidence and high curability after definitive surgery alone. Radiotherapy may be considered an adjuvant treatment in cases of close or positive surgical margins. One study highlighted the potential benefit of adjuvant radiotherapy in patients with head and neck IMT with unfavorable factors for malignant transformation [65]. These included tumor size >4.4 cm, tumors in the maxillary sinus, and a preoperative neutrophil/lymphocyte ratio greater than 1.958. The authors retrospectively reviewed 45 patients with head and neck IMT who underwent radical surgical resection. Twenty of them also received postoperative radiotherapy. Interestingly, postoperative radiotherapy did not benefit the whole group, but in patients with malignant transformation, postoperative radiotherapy significantly improved overall survival. In addition, postoperative radiotherapy improved overall survival in patients with head and neck IMT at high risk of malignant transformation.

Adjuvant radiotherapy may be a valuable treatment option even in the presence of contraindications to adjuvant systemic treatment. Lisi et al. described the case of a 7-week pregnant woman who developed ALK-positive IMT localized to the trachea [167]. The patient terminated her pregnancy and underwent total surgical resection with close margins (<1 mm). She refused adjuvant ALK inhibitors due to the risk of ovarian suppression. She therefore received 45 Gy in 25 fractions post-operatively with excellent tolerability and no evidence of recurrence at longer follow-up.

### Definitive radiotherapy

Following an individualized approach, definitive radiotherapy combined with systemic treatment or steroids can be offered to patients with partially resected or unresectable disease. Zhu et al. presented the results of an analysis of 13 patients with IMT of the paranasal sinuses and nasopharynx [168]. Ten of these patients received definitive radiotherapy in various combinations with partial surgery, chemotherapy, and steroids. The majority of patients had stable disease after treatment. Another report showed a case of a patient with IMT of the head and neck that invaded maxillary sinus [169]. Due to the unresectability of the tumour, he underwent definitive radiotherapy with a dose of 60 Gy in conventional 2 Gy fractions over 6 weeks, concurrently with oral prednisolone. Follow-up magnetic resonance imaging performed 2 months after treatment confirmed a complete radiological response of the tumour. Another case study focuses on a patient with IMT leading to severe stenosis of the left pulmonary artery who was also receiving immunosuppressive treatment for perinuclear antineutrophil cytoplasmic antibody vasculitis. The patient received radiotherapy with a total dose of 45 Gy over five weeks and has been followed for over seven years post-treatment without persistent toxicity [170].

Maire et al. reported the case of a 38-year-old man who presented with headache, right-sided exophthalmia, and paralysis of the right 6th nerve [171]. MRI revealed a large skull base tumor extending into the sella turcica, right cavernous sinus, and sphenoidal sinus. Biopsy confirmed the presence of IMT. Despite three months of corticosteroid treatment, there was no improvement. The patient then received 20 Gy in 10 fractions over 12 days. Three months after radiotherapy, there was a complete clinical response. A near-complete radiological response was observed at six months. Two years after treatment, there is no evidence of local recurrence.

### Low-dose radiotherapy

IMT seems to be radiosensitive. As we have shown above, most of the reports use moderate total doses in conventional fractions, up to 45 Gy, and show good local response. However, selected papers highlighted even higher radiosensitivity, similar to follicular and MALT lymphomas. One patient with recurrent bilateral pulmonary IMT was treated with low-dose radiotherapy, namely 4 Gy in 2 fractions [172]. Three months after the initial scan, imaging showed a partial response of the lesion, and a follow-up CT scan six months later, without any additional treatment, showed an almost complete response. Since then, the patient has undergone several CT scans, which have consistently shown a stable lesion in the lingula, with intermittent changes in ground-glass opacities in various locations. In another aforementioned study, 20 Gy in 10 fractions enabled near-complete response and long-term local control [171].

## Systematic Treatment

### Neoadjuvant and adjuvant chemotherapy

Currently, there are no indications for the administration of neoadjuvant systemic therapy to patients with IMT; As mentioned, radical resection remains the only established treatment for locally advanced tumors. In the systemic therapy of the IMF, classic chemotherapy was mostly used, currently being replaced by ALK inhibitors, steroids, or non-steroidal anti-inflammatory drugs (NSAIDs). There are some historical reports on neoadjuvant chemotherapy as an initial treatment [11]. However, when inhibitors of receptor tyrosine kinases are available, multiple case reports indicate the usefulness of neoadjuvant-targeted therapy in this indication [173,174]. First-generation inhibitors, such as crizotinib, and next-generation inhibitors, such as lorlatinib, are proposed treatment options. Importantly, lorlatinib has shown efficacy in overcoming resistance associated with first-generation inhibitors, leading to a significant reduction of tumor burden [175]. This therapeutic strategy can potentially decrease the size of the tumor in critical organs, facilitating radical resection while preserving organ function. Additionally, it may make tumors initially thought to be inoperable and now suitable for surgery. Furthermore, mainly due to the clinical presentation of this cancer, there is data on the neoadjuvant use of cyclooxygenase-2 inhibitors (COX-2) and steroids. Case reports showed that a selective COX-2 inhibitor, meloxicam 10 mg/d, in combination with prednisolone 45 mg/d, allowed it to shrink the tumor from 47 to 27 mm, with the clinical benefit of organ-sparing surgery [176].

However, these regimens are used more frequently in clinical practice in the adjuvant setting, particularly when surgical margins are not negative. The use of postoperative systemic treatment in the form of ALK inhibitors, steroids, or NSAIDs has been shown to reduce the risk of recurrence and improve patient prognosis [177,178]. In a retrospective analysis, adjuvant treatment was administered to 5 patients with chemotherapy regimens consisting of methotrexate and vincristine or ifosfamide, carboplatin, etoposide, and taxol. Local recurrences were observed only in the group of patients with incomplete resection without adjuvant therapy [162]. Furthermore, a combination of chemotherapy and anti-inflammatory drugs showed efficacy in this indication. Administration of 4 courses of ifosfamide (9 g/m^2^ total per cycle) and adriamycin (75 mg/m^2^ total per cycle) followed by 2 complementary courses of ifosfamide (6 g/m^2^ total per cycle) with a 3-week interval between courses accompanied by ketorolac allowed achieving a reduction in tumor size from 40 mm × 13 mm × 37 mm to 12 mm × 5 mm × 11 mm [179]. Multiple case reports demonstrated the benefit of adjuvant use of ALK inhibitors, especially clinical benefit with reduction of the most severe symptoms among patients [134,180,181]. Among the eight patients evaluated for the effect of crizotinib, all responded to the drug, achieving a partial response (PR) or complete response (CR), crizotinib was administered perioperatively at a dose (280 mg/m^2^/dose twice a day orally) [182]. Recently, an analysis of ALK inhibitors in IMT showed that among 29 evaluated patients, 26 patients experienced CR or PR (both neoadjuvant and adjuvant treatment) after initial treatment [183]. However, these results are relevant for patients with ALK-positive tumors [184]. Contradictory results were achieved in the Casanova et al. study indicating a good response to surgery, with a possible long-term response to systemic therapy in the event of recurrence [185]. Due to inconclusive results and a limited study group, the usefulness of perioperative treatment remains to be determined.

### Systematic treatment of advanced/metastatic disease

Systemic therapy is specifically recommended for IMT patients who experience unresectable, advanced, or metastatic disease. There is a lack of consensus on the most optimal treatment regimen for these individuals.

### Chemotherapy

Conventional treatment for IMT is the same as in other non-small cell sarcomas, predominantly with anthracycline-based regimens, however, there are no specific guidelines for that subtype. A report from nine European sarcoma reference centers evaluated 38 patients with IMT, of whom 25 received various chemotherapy regimens. Of the 38 patients, 25 (66%) received anthracycline-based chemotherapy, 13 (34%) underwent methotrexate with or without vinorelbine/vinblastine (MTX-V) chemotherapy, and 10 (23%) were treated with alternative regimens, such as oral cyclophosphamide and docetaxel/gemcitabine-8 patients treated for localized and 17 for advanced disease. The overall response rate (ORR) was 10/21 (47.6%). For patients with localized disease, the median follow-up was 70.8 months, and the median recurrence-free survival (RFS) and the median overall survival (OS) were not reached. For patients with advanced disease, the median progression-free survival (PFS) was 6.3 months and the median overall survival (OS) was 21.2 months at the same follow-up. Anthracycline-based chemotherapy showed similar efficacy to methotrexate chemotherapy (ORR 53.8%), furthermore, both regimens showed significantly greater activity compared to the standard results achieved in this group of sarcomas [186]. However, more side effects of therapy were observed in the group treated with anthracyclines. Grade (G)3 or G4 adverse events were observed in 7/25 (28%) patients, while for other chemotherapeutics, G3 or G4 toxicity events occurred in 2 patients (15%). No correlation was observed between treatment outcome and ALK status [187]. These findings were confirmed in the pediatric population, as evidenced by the European Pediatric Soft Tissue Sarcoma Study Group (EpSSG) study, which demonstrated 4 complete responses (CR), 8 partial responses (PR), and 5 stable diseases (SD), resulting in a response rate of 63%. The regimen used most frequently in this study was the vinblastine-methotrexate combination [185].

### Targeted therapy

Genetic abnormalities identified in IMT serve not only as the basis for the diagnosis and understanding of the pathophysiology of the tumor but also constitute a crucial therapeutic target. First, introduced in the research of non-small cell lung cancer (NSCLC), ALK inhibitors have been applied in the treatment of IMT with good efficacy. Presently, there are three generations of these inhibitors: crizotinib, a first-generation drug; ceritinib, alectinib, ensartinib, and brigatinib, a second-generation drug; and lorlatinib, a third-generation drug [188]. In 2010, the initial report documenting the efficacy of crizotinib in the context of IMT emerged, coinciding with analogous reports of its application in lung cancer. Two patients were subjected to crizotinib therapy: one characterized by rearrangement of ALK-RANBP2 and another without detectable rearrangements of ALK. Following administration of an initial dose of 200 mg daily, Patient 1 achieved a PR according to the Response Evaluation Criteria in Solid Tumors (RECIST), marked by a 53% reduction in the size of the target lesions. Despite the appearance of new lesions, the therapeutic regimen persisted and the dose was subsequently increased to 250 mg daily, resulting in a sustained response to treatment. On the contrary, Patient 2, despite immediate initiation at the maximum tolerated dose, remained resistant to treatment [134]. After that, multiple case reports showed the varying effectiveness of all generations of ALK inhibitors, depending on the patient’s mutation status and additional interventions used, these case reports are summarized in Table 2.

These findings were substantiated by the results of the phase 2 clinical trial EORTC 90101 CREATE (NCT01524926), focusing on advanced and inoperable IMT [213]. The study enrolled individuals 15 years and older with advanced, inoperable IMT, regardless of previous systemic or local treatments. The patients were classified into ALK positive and negative cohorts based on FISH and/or IHC. The prescribed regimen included a twice-daily dose of 250 mg of crizotinib, the primary endpoint being ORR. The results revealed that 50% (6/12) of patients with ALK-rearranged tumors and 14% (1/7) of patients negative for ALK exhibited an objective response to crizotinib. Among the ALK-positive group, two out of six patients achieved CR [213]. A recent update on long-term efficacy update in this trial demonstrated even more promising results, with an ORR of 66.7%, a median PFS of 18.0 months (95% CI 4.0–NE), and a 3-year overall survival rate (OS) of 83.3% (95% CI 48.2–95.6) in ALK-positive IMT [214]. A retrospective analysis of 30 patients treated with crizotinib showed an ORR of 81.3% and a disease control rate of 87.5% [215]. In particular, crizotinib demonstrated efficacy in the pediatric population with an acceptable toxicity profile. A phase 1 pediatric trial (NCT00939770) included participants aged >12 months and <22 years, with a specific focus on evaluating the efficacy, tolerability, and safety in 7 patients with inflammatory IMT. Within this group, PR was observed in 3 patients, leading to the establishment of a dose of 280 mg/m^2^ twice daily [216]. Subsequent stages of the study expanded the participant pool to 14 individuals, resulting in an ORR of 86% (95% CI, 57 to 98), with CR observed in 36% (5 of 14) cases and PR in 50% (7 of 14) cases [217]. Based on that, on 14 July 2022, the Food and Drug Administration (FDA) approved crizotinib for the treatment of advanced IMT positive for ALK [218].

As appropriately acknowledged in NSCLC and confirmed by the findings of select case reports outlined in Table 2, resistance to first-generation ALK inhibitors is frequent. This resistance, whether primary or acquired during treatment was reported [219]. Sequential therapy, similar to that employed in NSCLC, may also confer benefits in patients with IMT [66]. Consequently, successive generations of inhibitors are undergoing investigation. Second-generation ALK inhibitors succeeded in overcoming resistance as presented in several case reports. Furthermore, alectinib was evaluated among 7 patients with ALK-rearranged tumors, with 3 patients with IMT. One patient achieved CR and one patient achieved PR after switching from crizotinib to a second-generation inhibitor; the overall PFS for this trial was 8.1 months [220]. In phase 1 clinical trial involving pediatric patients diagnosed with ALK-positive IMT, ceritinib demonstrated an ORR of 70%. In particular, a patient who had received prior treatment with crizotinib achieved CR when treated with ceritinib [221]. In a phase 2 trial that evaluated adult patients (NCT02465528) who had received 1 prior systemic therapy, ceritinib was administered 750 mg/day. Among the 4 patients enrolled in IMT, 3 achieved PR and ORR which was 75.0% (95% CI 19.4–99.4) [222]. Furthermore, a clinical trial is currently underway assessing the efficacy of brigatinib in IMT (NCT04925609).

### Immunotherapy

Information on the use of immunotherapy in the form of checkpoint inhibitors in IMT is very limited, and the evidence is based on single case reports. Programmed cell death ligand 1 (PD-L1) expression in IMT affects approximately 69% of tumors and 80% of tumors have PD-L1(+) immune cells. Recurrent/metastatic tumors (80%) and ALK (88%) were PD-L1(+) [223]. The use of immunotherapy in the form of toripalimab 240 mg for 1 cycle followed by 6 cycles of sintilimab 200 mg (both programmed cell death protein 1-PD-1 and PD-L1 inhibitor) allowed achieving PR after 7 cycles of treatment and CR after 17 cycles in a patient with nasopharyngeal IMT and the recurrence in the skull base, slope and pterygoid sine [224]. In a study of the effectiveness of immunotherapy in various histological subtypes of sarcomas, out of 26 included patients, only 3 responded to treatment, including a patient with IMT. She was treated with nivolumab monotherapy as positive PD-1, but with negative PD-L1 status, PR was achieved, defined as at least 30% regression in target tumor burden [225]. Considering the poor responses of patients with ALK-negative tumors, immunotherapy may prove to be an important therapeutic option.

### Other regimens

Due to the heterogeneous genetics of IMT and various gene fusions, it is necessary to introduce other targeted therapies. The presence of ROS1 fusion genes has been proven; Reports on the therapy of these patients are poorer, but case reports showed the efficacy of standard ALK inhibitors in this case [210,226,227]. As mentioned above in the Genetics section, IMT may harbour neurotrophic tropomyosin receptor kinase (NTRK) gene rearrangements. Recently, a report from three phases 1-2 clinical trials resulted in the approval of entrectinib as an inhibitor of tropomyosin receptor kinase (TRKi) with antitumor activity against tumors positive for NTRK gene fusion. Among the trials (ALKA-372-001, STARTRK-1, and STARTRK-2) 13 patients with sarcomas were identified and six (46%; 19–75) had a response to treatment [228]. The overall mPFS of this analysis was 11 months (95% CI 8.0–14.9) and the mOS was 21 months (95% CI 14.9–NE) [229]. Interestingly, the authors did not observe differences in drug effectiveness depending on the fusion partner. In the pediatric population, TRKi resulted in achieving a CR in 20 out of 27 evaluated patients. Typically, these inhibitors were combined with other therapies such as surgery or radiotherapy. The five-year OS in this study was 88% (95% CI, 73.5–100) [230]. Larotrectinib is a related drug that has also shown effectiveness in patients with NTRK-rearranged tumors, however, no specific results were reported in the IMT subgroup [231]. Furthermore, a neoadjuvant larotrectinib demonstrated its efficacy in NTRK-altered tumors, resulting in the patient attaining a CR when combined with radical surgical intervention facilitated by the regimen [232]. In xenograft models representing an aggressive subtype of IMS (epithelioid IMS with RANBP2-ALK rearrangement), the concurrent inhibition of CD30 and ALK demonstrated efficacy, suggesting the potential relevance of new targets in this indication; however, more research is needed [233]. A phase 1 clinical trial ALKOVE-1 (NCT05384626) is currently underway for NVL-655, a newly developed brain-penetrant ALK selective inhibitor designed to overcome limitations observed with currently available regimens.

## Prognostic Factors

Regarding the prognosis of IMT, most patients who undergo radical treatment show no recurrence or metastasis. In the pediatric population, studies report 5-year event-free survival (EFS) of 82.9% and OS of 98.1% [185], figures consistent with the findings in studies involving adults [178]. However, the exploration of prognostic and predictive factors in IMT remains limited, with most studies focusing primarily on establishing correlations between the morphological and genetic characteristics of the tumor and its clinical behavior. The most well-established prognostic factor for people with IMT is the feasibility of radical resection. Evidence shows that most recurrences occur in patients who have undergone nonradical surgery [162,234–236]. This correlation is directly related to tumor size, with patients who harbor tumors larger than 6.5 cm exhibiting a poorer prognosis [55]. A prognostic factor for recurrence is identified at a tumor size of approximately 11 cm, although these specific values may vary slightly between studies [11,237]. The impact of patient age remains somewhat unclear, some studies suggest a more aggressive course of the disease in older patients, while others indicate a higher risk of metastatic disease in younger individuals [11,55]. In a study that suggested a worse prognosis among younger patients, this conclusion was due to additional factors. Younger patients presented with large, multinodular tumors in key locations that often prevented resection and indirectly contributed to prognosis [11]. Conversely, a study with opposing conclusions found that young age was a factor of better prognosis in multivariate analysis (*p* = 0.027) [55]. However, drawing clear conclusions from these analyses is difficult due to the different patient groups compared and additional factors that may have influenced the analysis, particularly with regard to age. Pathological findings correlated with a poor prognosis are mainly histological atypia represented by the occurrence of necrosis, lymphovascular invasion, high mitotic activity, increased cellularity, and invasive border, along with myxoid intercellular content, ganglion-like cells, and giant cells [237]. Reports on ALK expression in IHC are contradictory; however, some studies implicated more favorable outcomes in patients with ALK(+) tumors [53,234]. However, these results mainly related to the risk of metastatic disease, suggesting that ALK-negative tumors were more likely to correlate with metastatic disease [53]. Additionally, a study in the pediatric population found a correlation between expression and radical resection, but only in patients with unresectable disease [234]. On the contrary, analysis of a pediatric cohort of patients showed that both overexpression of ALK and ALK mutation status did not affect EFS [178]. Also, Casanova et al. reports confirmed these results, since EFS was the same in the group of ALK-negative and positive tumors [185]. Therefore, it appears that neither mutations nor ALK expression have a significant impact on the prognosis of patients. In terms of other genetic alterations, the impact on prognosis is rarely examined, but individual reports can be concluded that, among others, the results of treatment in patients with ROS1 gene reaction are particularly positive [238]. eIMT, a markedly aggressive form of inflammatory myofibroblastic tumor, distinguished by epithelioid morphology, significant neutrophilic infiltrate, and nuclear membrane staining of ALK with an associated ALK rearrangement, has an especially unfavorable prognosis. Most cases are correlated with RANBP2 gene fusions, but EML4-ALK and VCL-ALK fusions are also possible [143,239]. Nevertheless, the exact cause of this subtype’s more aggressive behavior remains to be elucidated.

## Conclusions

Our understanding of IMT pathology is limited due to the rarity of the disease and the difficulty in assembling a significant number of cases. However, recent advances in diagnostic techniques, such as NGS and computational analysis, show promise in clarifying specific aspects of IMT biology. This will facilitate the identification of tailored treatment approaches. Managing IMT presents significant clinical complexities and is an area where personalized medicine is at its peak. Determining the most effective TKI strategies for ALK-positive IMT remains an ongoing effort. In addition to ALK targeting, comprehensive data on the efficacy of alternative TKIs, chemotherapy, and immunotherapy is critical. The advancement of our knowledge in these areas has the potential to further refine the understanding and clinical management of IMT in the future.

## Data Availability

Not applicable.
